# Phosphodiesterase-4 Inhibitors for the Treatment of Inflammatory Diseases

**DOI:** 10.3389/fphar.2018.01048

**Published:** 2018-10-17

**Authors:** Heng Li, Jianping Zuo, Wei Tang

**Affiliations:** ^1^Laboratory of Anti-inflammation, Shanghai Institute of Materia Medica, Chinese Academy of Sciences, Shanghai, China; ^2^School of Pharmacy, University of Chinese Academy of Sciences, Beijing, China; ^3^Laboratory of Immunopharmacology, State Key Laboratory of Drug Research, Shanghai Institute of Materia Medica, Chinese Academy of Sciences, Shanghai, China; ^4^Open Studio for Druggability Research of Marine Natural Products, Pilot National Laboratory for Marine Science and Technology, Qingdao, China

**Keywords:** phosphodiesterase-4, psoriasis, atopic dermatitis, inflammatory airway diseases, inflammatory bowel disease, roflumilast, apremilast, crisaborole

## Abstract

Phosphodiesterase-4 (PDE4), mainly present in immune cells, epithelial cells, and brain cells, manifests as an intracellular non-receptor enzyme that modulates inflammation and epithelial integrity. Inhibition of PDE4 is predicted to have diverse effects via the elevation of the level of cyclic adenosine monophosphate (cAMP) and the subsequent regulation of a wide array of genes and proteins. It has been identified that PDE4 is a promising therapeutic target for the treatment of diverse pulmonary, dermatological, and severe neurological diseases. Over the past decades, numerous PDE4 inhibitors have been designed and synthesized, among which roflumilast, apremilast, and crisaborole were approved for the treatment of inflammatory airway diseases, psoriatic arthritis, and atopic dermatitis, respectively. It is regrettable that the dramatic efficacies of a drug are often accompanied by adverse effects, such as nausea, emesis, and gastrointestinal reactions. However, substantial advances have been made to mitigate the adverse effects and obtain better benefit-to-risk ratio. This review highlights the dialectical role of PDE4 in drug discovery and the disquisitive details of certain PDE4 inhibitors to provide an overview of the topics that still need to be addressed in the future.

## Introduction

Inflammation underlies the pathogenesis of various human diseases, which includes infection, immune-mediated disorders, metabolic disturbance, neurodegeneration, and cancer. Inflammatory diseases affect a certain population worldwide and possess extremely complicated pathogenic mechanisms (Kazatchkine and Kaveri, 2001). To date, numerous therapeutic strategies have emerged in the treatment of inflammatory diseases (Tabas and Glass, 2013; Siebert et al., 2015). Though the non-steroidal anti-inflammatory drugs (NSAIDs) and corticosteroids have made tremendous contributions for inflammation intervention, the serious long-term adverse effects and the multiple manifestations of diseases drive some patients away from these therapeutic options (Hart and Huskisson, 1984). Hence, there remains a great need for the discovery of novel therapeutic drugs for controlling inflammation (Uguccioni et al., 2017).

Cyclic guanosine monophosphate (cGMP) and cyclic adenosine monophosphate (cAMP) function as the fundamental second messengers in the regulation of multiple cellular metabolisms. Phosphodiesterases (PDEs), consisting of 11 families (PDE1–PDE11), are available for the degradation of cyclic nucleotides (Kumar et al., 2013). Distributions of PDE subfamilies are diverse in different cells and tissues, which may provide a substantial support for their pharmacological research in the field of inflammation, cognition, lipogenesis, proliferation, apoptosis, and differentiation. The cAMP-specific PDE4 is highly expressed in the brain, cardiovascular tissues, smooth muscles, keratinocytes, and immunocytes (including T cells, monocytes, macrophages, neutrophils, dendritic cells, eosinophils) (Chiricozzi et al., 2016). The inhibition of PDE4 can elevate the intracellular level of cAMP and subsequently modulate the inflammatory responses and maintain the immune balance (Maurice et al., 2014).

**Figure 1:**
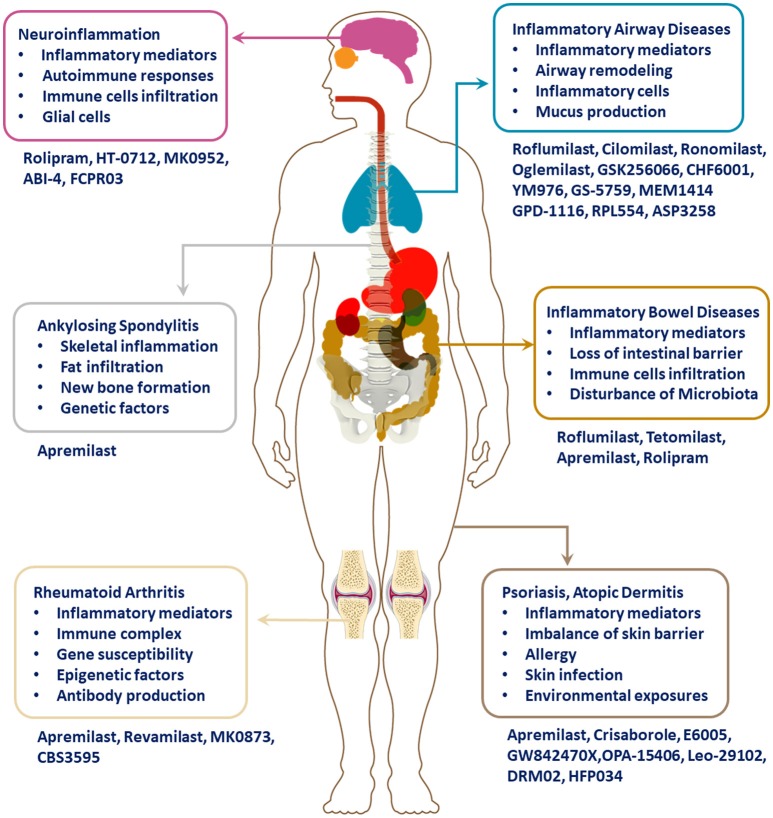
Pathological manifestations and PDE4 inhibitors in inflammatory diseases.

Targeting PDE4 has been verified as an effective therapeutic strategy for inflammatory conditions, including asthma, chronic obstructive pulmonary disease (COPD), psoriasis, atopic dermatitis (AD), inflammatory bowel diseases (IBD), rheumatic arthritis (RA), lupus, and neuroinflammation (as shown in Figure 1). Through great efforts, roflumilast, apremilast, and crisaborole were approved in succession for the treatment of inflammatory airway or skin diseases. Moreover, a series of novel PDE4 inhibitors have also been developed for the regulation of inflammation, and they showed satisfactory therapeutic efficacies. This review summarizes the chemical skeleton, pharmacological and clinical details of licensed PDE4 inhibitors in the process. Nausea, emesis, gastrointestinal effects, and other adverse effects have largely impeded the clinical application, which is attributed to PDE4 inhibition in the unexpected tissues. Hence, more efforts and emphasis are required to balance efficacy with minimizing adverse effects in the long and bumpy road to the development of more innovative drugs.

## Mode of action: PDE4 inhibition in the regulation of inflammation

Phosphodiesterase-4 was found to be a dramatic downstream component of the β-adrenoceptor and N-methyl-D-aspartic acid receptor (NMDAR) mediated signaling pathway and also related to 5-hydroxytryptamine (5-HT) receptor (O'Donnell and Zhang, 2004). Receptors for activated C kinase 1 (RACK1) and A-kinase-anchoring proteins (AKAPs) and proteins that contain SH3 domains act as the interacting proteins that affect the intracellular localization and function of PDE4 (Maurice et al., 2014). Delicate combinations of cyclic nucleotide modulators, including protein kinase A (PKA), cyclic nucleotide-gated ion channels, and exchange factors directly activated by cAMP (Epac) with PDE4 contribute to the formation of cAMP signalosomes via protein-protein interactions, which showed multiple effects on cell proliferation, differentiation, and immune responses (Motte et al., 2017).

**Figure 2:**
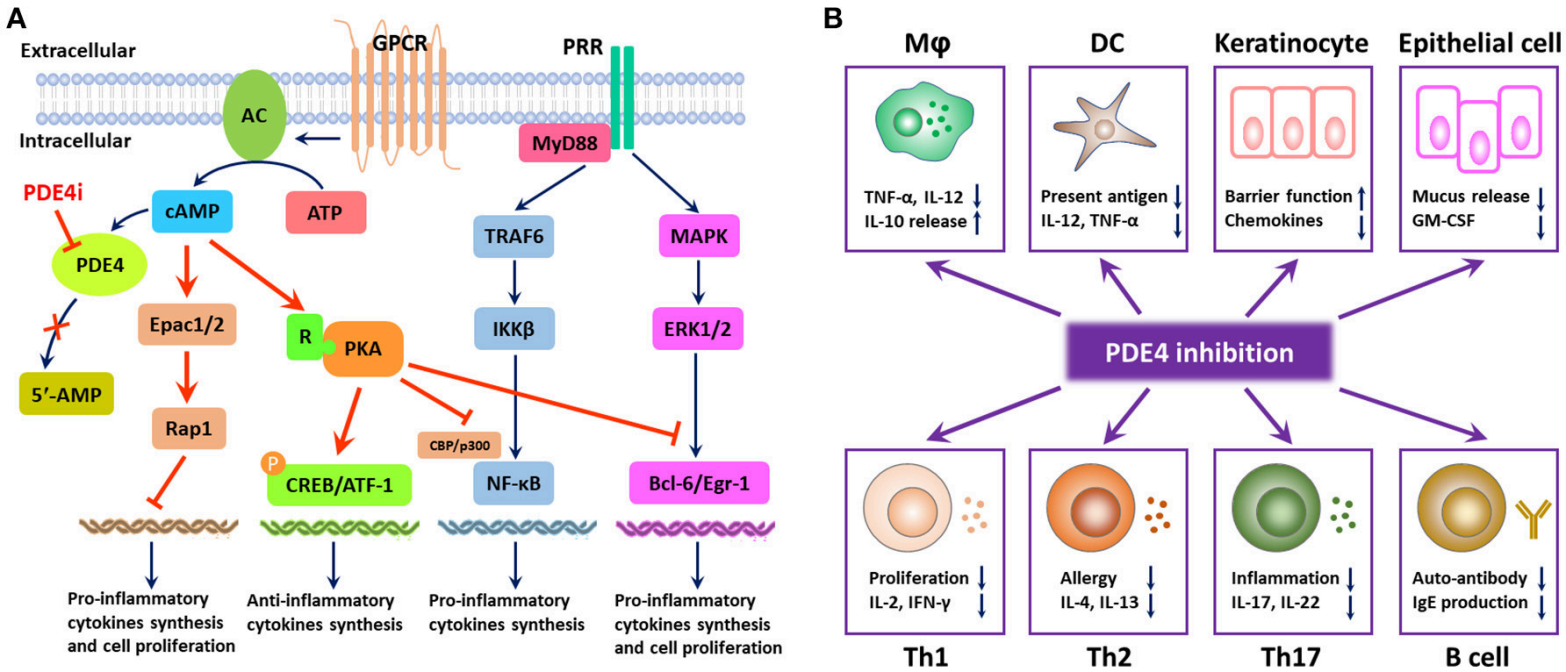
Mode of PDE4 inhibition in the regulation of inflammatory responses. **(A)** PDE4 regulates the production of pro-inflammatory and anti-inflammatory cytokines and cell proliferation via the degradation of cAMP. PDE4 inhibition leads to the accumulation of intracellular cAMP, which can activate protein kinase A (PKA) and the exchange protein 1/2 activated by cAMP (Epac1/2). PKA activation results in the phosphorylation of cAMP-responsive element binding protein (CREB) and activating transcription factor 1 (ATF-1), leading to the increase in anti-inflammatory cytokines. The transcriptional activity of NF-κB can be regulated by PKA activation through the modulation of its interaction with CREB binding protein (CBP) or p300; meanwhile, PKA activation can interfere with the synthesis of B-cell lymphoma 6 protein (Bcl-6)-mediated pro-inflammatory cytokines and the proliferation of immune cells. Compartmentalization of intracellular cAMP contributes to the Epac signalosome of transcription factors, small GTPases (Rap1), which serves as a promising alternative mechanism to target inflammation and proliferation. **(B)** PDE4 inhibition has a broad spectrum of anti-inflammatory effects. Owing to the distribution of PDE4 in the human body, PDE4 inhibition can inhibit inflammatory responses from macrophages, DCs, Th1, Th2, and Th17 cells, increase the production of anti-inflammatory cytokines from macrophages, and interfere with the phenotype and function of B cells as well. Moreover, PDE4 inhibition can also promote the barrier function of keratinocytes and epithelial cells via suppression of the inflammatory mediator production.

Increasing evidence demonstrated that patients who suffered from inflammatory diseases showed higher expression of PDE4 than the healthy individuals (Schafer et al., 2016). There are four subtypes of PDE4, namely PDE4A–PDE4D, which are highly specific for cAMP degradation but not for cGMP. Inhibition of PDE4 results in the accumulation of intracellular cAMP and subsequently activates PKA, cyclic nucleotide-gated ion channels, and Epac1/2. These are involved in the regulation of pro-inflammatory and anti-inflammatory cytokines synthesis, activation of T cells, neutrophil degranulation, performance of antigen-presentation, and epithelial integrity via initiation of multiple downstream elements (as shown in Figure 2A). Release of catalytic subunit from regulatory subunit upon PKA activation could subsequently increase the phosphorylation of cAMP-responsive element binding protein (CREB), activating transcription factor 1 (ATF-1) and cAMP responsive element modulator (CREM) and recruit the CREB binding protein (CBP) or the homologous protein p300, leading to the reduction of inflammatory cytokines and the increase of anti-inflammatory cytokines (Schafer, 2012). A previous study demonstrated that the transcriptional activity of classic nuclear factor kappa-light-chain-enhancer of activated B cells (NF-κB) can be stimulated upon the phosphorylation of p65 on Ser276 by PKA (Christian et al., 2016). The CBP/p300 is closely associated with NF-κB p65, and PKA activation could regulate the transcriptional activity of NF-κB through the modulation of its interaction with CBP/p300 without IκBα degradation or NF-κB DNA binding activity, which results in the downregulation of inflammatory responses (Zhong et al., 1998; Schafer, 2012). Additionally, PKA activation could interfere with B-cell lymphoma 6 protein (Bcl-6)-mediated synthesis of pro-inflammatory cytokines and proliferation of immune cells (Page, 2014; Hernández-Flórez and Valor, 2016). Activation of Epac1/2 in the wake of cAMP elevation serves as a promising alternative mechanism to target inflammation and proliferation (Lehrke et al., 2015). Compartmentalization of intracellular cAMP in space and time contributes to the Epac signalosome of transcription factors, small GTPases (Rap1), which do well in the optimization of the treatment of inflammatory airway diseases, renal failure, vasculature disturbance, and neuroinflammation (Schmidt et al., 2013).

Given the role of cAMP in diverse physiological metabolisms in various kinds of cells, cAMP elevation following PDE4 inhibition is closely associated with the suppression of the overactivity of immune responses or intermediates (as shown in Figure 2B). Accumulating research indicated that PDE4 inhibition could modulate both innate and adaptive responses. Inhibition of PDE4 showed regulatory activities in macrophages, neutrophils, monocytes, and dendritic cells (Crilly et al., 2011; Schafer, 2012). In addition, PDE4 inhibition showed excellent effects on T cell receptor (TCR)-induced activation of T cells, manifesting in the reduction of release of cytokines and chemokines from T helper-1 (Th1), Th2, and Th17 cells (Sakkas et al., 2017), whereas PDE4 inhibition might have little effect on the phenotype and function of B cells (Schafer et al., 2014). Furthermore, elevated cAMP in keratinocytes and epithelial cells could also inhibit the inflammatory responses and regulate the cell growth and barrier functions (Page, 2014).

## Approved PDE4 inhibitors for the treatment of inflammatory diseases

Some breakthroughs have been achieved in the discovery of therapeutic PDE4 inhibitors, recently, for patients with inflammatory diseases. Rolipram was the first prototypic PDE4 inhibitor that was identified in the early 1990s by Schering AG as an excellent antidepressant drug; however, its therapeutic window was too narrow and clinical trials were inseparable from high rates of adverse events, especially nausea and vomiting (O'Donnell and Zhang, 2004). On top of rolipram, more attractive PDE4 inhibitors have been uncovered, among which roflumilast, apremilast, and crisaborole were approved for the market, consecutively (Sakkas et al., 2017).

### Roflumilast approved for the treatment of COPD and asthma

Currently affecting more than 200 million individuals worldwide, COPD manifests as a progressive damage to the pulmonary integrity with various clinical symptoms and complicated pathophysiological mechanisms (Caramori et al., 2017; Benton et al., 2018). Abnormal immune responses and severe inflammation in the lung airways are closely related to the initiation and progression of COPD. Infiltration of innate and adaptive inflammatory cells into the local lung tissue is considered as the fundamental pathogenic factor in COPD patients. Furthermore, available evidence indicated that the neutrophil-to-lymphocyte ratio (NLR) might predict the progression and outcomes of COPD (Paliogiannis et al., 2018). Asthma is another refractory inflammatory airway disease, which is characterized by bronchial hyperreactivity, mucus production, airway narrowing, and remodeling, along with explosion of inflammatory cells, especially neutrophils (Gao et al., 2017; Benton et al., 2018; Castro-Rodriguez et al., 2018). Undoubtedly, COPD and asthma patients share similar clinical phenotypes, and it is difficult to distinguish asthma from COPD, particularly when they coexist in elderly patients (Tochino et al., 2017). In the past decades, PDE4 inhibitors used in the treatment of COPD and asthma have been dramatically attracting the interests of pharmacists. Inhibition of PDE4 suppresses airway inflammation extremely and relaxes smooth muscle via the elevation of the level of cAMP.

**Figure 3:**
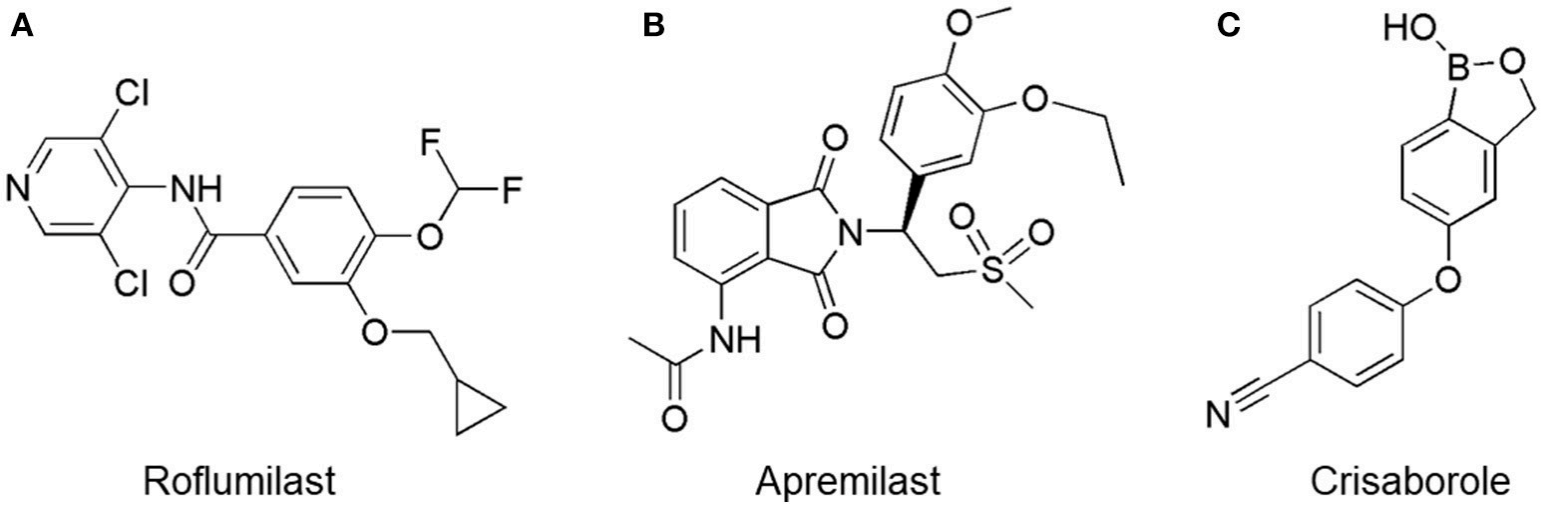
Approved PDE4 inhibitors for the treatment of inflammatory diseases. **(A)** Roflumilast was approved in the EU (2010) and USA (2011) for the treatment to reduce the risk of COPD exacerbations in patients with severe COPD associated with chronic bronchitis and a history of exacerbations. **(B)** Apremilast was approved in USA (2014) for adult patients with active psoriatic arthritis and patients with moderate-to-severe plaque psoriasis who were candidates for phototherapy or systemic therapy. **(C)** Crisaborole was approved in USA (2016) for topical treatment of mild-to-moderate atopic dermatitis in patients aged 2 years and older.

Oral administration of roflumilast (trade name Daxas, Figure 3A) was licensed for the treatment of severe COPD and asthma symptoms in the EU and USA in 2010 and 2011, respectively, which marked the first approved PDE4 inhibitor. Roflumilast has been investigated to be a potent anti-inflammatory drug in the regulation of airway inflammation (Cazzola et al., 2016; Kawamatawong, 2017). *In vitro*, roflumilast inhibited PDE4 activity (IC_50_ = 0.8 nM) in human neutrophils with high selectivity and, therefore, showed excellent anti-inflammatory potential in fMLP-induced leukotriene B_4_ (LTB_4_) and reactive oxygen species (ROS) formation in human neutrophils, lipopolysaccharides (LPS)-induced tumor necrosis factor α (TNF-α) synthesis in monocytes, dentritic cells as well as cytokines production in anti-CD3/CD28-stimulated CD4^+^ T cells (Hatzelmann and Schudt, 2001; Bros et al., 2016). Moreover, roflumilast markedly suppressed the production of inflammatory mediators in macrophages via the induction of heme oxygenase (HO-1) expression and the inhibition of NF-κB, p38 MAPK, and JNK activation (Kwak et al., 2005a,b). Accumulating studies showed that mitophagy mediated the death of pulmonary epithelial cell under cigarette smoke extract (CSE) exposure, which contributed to the development and progression of emphysema. Roflumilast protected against CSE-induced cell death in Beas-2B cells, suggesting that roflumilast might do well in COPD treatment (Kyung et al., 2018). *In vivo* efficacies of roflumilast in airway inflammatory models suggested that roflumilast displayed bronchodilatory activity in spasmogen-challenged rats and guinea pigs and exhibited dose-dependent efficacy in airway hyperresponsiveness associated with eosinophilic inflammation evoked by ovalbumin (OVA) (Urbanova et al., 2017). Izikki et al. found that roflumilast could also inhibit hypoxia- and monocrotaline-induced severe pulmonary vascular remodeling and hypertension in rats (Izikki et al., 2009). Furthermore, roflumilast showed anti-inflammatory potentials in antigen-induced infiltration of immune cells and production of cytokines in bronchoalveolar lavage fluid of Brown Norway rats (Bundschuh et al., 2001). Airway remodeling is speculated to be associated with irreversible or partially reversible airway obstruction in asthma, which is well-known. Administration of roflumilast to OVA-challenged mice significantly inhibited airway inflammation, remodeling, and hyper-responsiveness (AHR) as well as the level of cytokines secreted by Th2 cells in the bronchoalveolar lavage fluids (Kim et al., 2016).

The study of pharmacokinetics demonstrated that the isoenzymes of cytochrome P450 (CYP450) played a vital role in converting roflumilast to its active metabolite, indicating that CYP450 inducers are not recommended for coadministration (Lipari et al., 2013). Clinical trials reported that roflumilast could suppress airway inflammation, improve the lung function of COPD patients, and reduce exacerbation of disease progression (Shen et al., 2018). A phase II/III, double-blind, randomized study showed that roflumilast significantly increased forced expiratory volume in 1 s (FEV_1_) and improved airway inflammation in asthma patients (Bateman et al., 2006). No significant neurological or cardiac toxicity was noted with roflumilast treatment. Nevertheless, roflumilast is known to have certain adverse effects that are significant enough to reduce compliance. Randomized clinical trials showed that adverse events (9.5%), including diarrhea, nausea, headache, weight loss, urinary tract infection, and psychiatric disturbance, were inevitable in clinical trials. In real-life clinical practice, it should be mentioned that high rate of adverse effects were much higher than those seen in randomized clinical trials (Gómez-Rodríguez and Golpe, 2017). In view of the relative balance between efficacy and safety, roflumilast indeed provides much more benefit than harm in patients, according to drug safety evaluation of medication under a correct education and administration strategy (Rogliani et al., 2016).

In addition to asthma and COPD, acute lung injury (ALI) and acute respiratory distress syndrome (ARDS) are characterized by transmigration and activation of immune cells and hypoxemia. Roflumilast improved the lung functions in a saline lavage-induced rabbit ALI (Kosutova et al., 2017) and alleviated pulmonary fibrosis and vascular remodeling in bleomycin-induced lung injury (Cortijo et al., 2009). Besides, ulcerative colitis (UC) is mainly characterized by inflammation and ulcers of the colon and rectum. A recent study showed that roflumilast attenuated the inflammation of dextran sulfate sodium (DSS)-induced UC in rats via the elevation of cAMP and the downregulation of inducible nitric oxide synthase (iNOS) expression (El-Ashmawy et al., 2018). Roflumilast (5 mg/kg/day) improved colon histologic score and prevented weight loss and decreased colon length. Moreover, roflumilast suppressed the production of inflammatory mediators and colon MPO activity (El-Ashmawy et al., 2018). Recently, a phase IIa, randomized, parallel group, double-blind, multi-center trial (NCT01856764) was conducted to assess the efficacy and safety of 0.5% roflumilast cream on AD patients. However, there were no significant changes in SCORAD (SCORing Atopic Dermatitis), TEWL (Transepidermal Water Loss), and pruritus after the topical application of roflumilast cream twice daily for up to 15 days; five patients reported adverse events, including application site pain, arthralgia, and nasopharyngitis (Zebda and Paller, 2018).

### Apremilast approved for the treatment of psoriasis and psoriatic arthritis

In the skin, PDE4 is primarily expressed in keratinocytes, neutrophils, Langerhans cells, and T cells, which contribute to the psoriatic plaque formation (Chiricozzi et al., 2016). Previous studies demonstrated that PDE4B and PDE4D mRNA level were higher in PBMCs from psoriasis as compared with normal individuals (Chiricozzi et al., 2016; Schafer et al., 2016). Owing to broad anti-inflammatory activities, PDE4 inhibitors have been investigated and applied for the treatment of various skin disorders or rheumatic diseases, such as psoriasis, psoriatic arthritis (PsA), and AD. Psoriasis is a chronic skin inflammatory disorder, which has an estimated global prevalence of 1–3% (Gisondi and Girolomoni, 2016). There are different kinds of phenotypes of psoriasis, including plaque-type, guttate, pustular, palmoplantar, and erythrodermic psoriasis (Greb et al., 2016). Unfortunately, prior to plaque formation, about 30% of the psoriasis patients developed PsA, which manifested additionally with joint inflammation and synovitis, distinct from RA, with enthesitis, dactylitis, or spinal involvement (Palfreeman et al., 2013). Psoriatic arthritis, characterized by progressive damage to peripheral and axial structures, is associated with genetic susceptibility, immune system imbalance, and environmental factors (Varada et al., 2014). Swelling, pain, or stiffness in one or more joints is commonly present in PsA patients. Generally, inflammation is considered as the underlying pathological process in psoriasis and PsA patients and NSAIDs, TNF-α inhibitors, interleukin-17 (IL-17) inhibitors, IL-12/IL-23 inhibitors, and PDE4 inhibitors have emerged in clinical treatment.

Apremilast is an orally administered PDE4 inhibitor that significantly inhibits inflammatory responses (Cauli et al., 2014; Christian et al., 2016; Smith, 2016). *In vitro*, apremilast was tested against endotoxin and superantigen-stimulated human PBMCs, bacterial peptide-primed polymorphonuclear cells (PMNs), immunonoglobulin and cytokines-activated NK cells, UVB-activated keratinocytes, human rheumatoid synovial cells, and LPS-stimulated RAW264.7 cells (Man et al., 2009; Schafer et al., 2010; Schafer, 2012). In the preclinical models of psoriasis and arthritis, oral application of apremilast significantly alleviated the epidermal thickness and abnormal proliferation and expression of TNF-α, HLA-DR, and ICAM-1 in the lesioned skin (Schafer et al., 2010). In addition, apremilast dramatically reduced the severity of arthritis in BALB/c mice and DBA/1J mice over 10 days post onset, with no evident side effects (McCann et al., 2010). Apremilast showed obvious effects on innate immunity and cellular immunity, especially the release of inflammatory mediators with a wide therapeutic index in ferret lung neutrophilia model, when compared with its gastrointestinal effects (Schafer et al., 2014). After great efforts, apremilast (brand name Otezla, Figure 3B) was approved in 2014 for treatment in adults who suffered from active PsA and moderate-to-severe plaque psoriasis (Cada et al., 2014; Varada et al., 2014; Chiricozzi et al., 2016). A pharmacokinetic study found that apremilast is absorbed well from the gut with independence of food intake and is mainly metabolized by CYP450 3A4, with minor contributions from CYP1A2 and CYP2A6, to about 23 metabolites clarified in plasma, urine, and feces (Bianchi et al., 2016). Apremilast exposure is unexpectedly reduced when coadministered with CYP450 inducers, such as rifampin, phenobarbital, carbamazepine, and phenytoin, which may result in the loss of therapeutic efficacy (Cada et al., 2014).

Unlike other topical treatments, apremilast shows wider applications and systemic impacts. However, some other adverse effects, including headache (5.9%), abdominal pain (2%), depression (1%), weight loss (10%), nausea (8.9%), diarrhea (7.7%), vomiting (3.2%), nasopharyngitis (2.6%), and upper respiratory tract infections (3.9%), were nonnegligible (Cada et al., 2014; Gooderham and Papp, 2015). To some extent, treatment with apremilast is related to an increased potential in depression or depression mood in the clinical trials. In the period of treatment, unwanted, or clinically significant weight loss should be evaluated without any delay and then discontinuation of apremilast therapy should be considered (Cada et al., 2014). Nausea and diarrhea that appeared in the first 2 weeks of apremilast treatment were predominantly light-to-mild, and the incidence rates of malignancies, additional severe infections, and major cardiac events were comparable with those in the placebo group. Furthermore, there were no evident clinical changes in lab markers and no novel cases of tuberculosis reported after apremilast therapy. Apremilast is contraindicated during pregnancy to avoid miscarriages and other pregnancy related problems. In one case about a 14-year-old adolescent patient, it was reported that he suffered from severe psoriasis symptoms that occurred in extremities, scalp, face, torso, and genitalia. After 1-month therapy with apremilast, significant improvement was observed, and he manifested no gastrointestinal effects when he took apremilast with food despite receiving the adult dose of apremilast. Briefly, the second available PDE4 inhibitor that can be oral administered, apremilast provides a satisfactory therapeutic window and may be well-tolerated in long-term exposure (Cauli et al., 2014). Nevertheless, extra clinical data are necessary to fully understand the therapeutic index of apremilast in children and adolescents with moderate-to-severe psoriasis (Smith, 2016).

In addition to psoriasis and PsA, apremilast has been investigated in other inflammatory conditions, including IBD, Behcet's Syndrome (BS), ankylosing spondylitis (AS), RA, frontal fibrosing alopecia, AD, and discoid lupus erythematosus, etc., in clinical phases (as shown in Table 1) (Abdulrahim et al., 2015). Apremilast exhibited therapeutic effects in the appearance and clinical outcomes of inflammatory disorders. In general, apremilast represents a breakthrough and a reward in the field of discovery for PDE4 inhibitors.

**Table 1:** Summary of ongoing clinical trials of apremilast in inflammatory diseases.

**NCT number**	**Indication**	**Intervention**	**Sponsor**	**Clinical stage**	**Primary endpoints**
NCT03422640	Frontal Fibrosing Alopecia	30 mg BID vs. placebo	Celgene Corp.	Phase IV	Clinical efficacy of up to 24 weeks therapy
NCT03239106	Itch	30 mg BID vs. placebo	Celgene Corp.	Phase II	Clinical efficacy of 16 weeks therapy
NCT03123016	Vitiligo	30 mg BID, combined with UVB treatment	Icahn School of Medicine at Mount Sinai	Phase II	Clinical efficacy of up to 64 weeks therapy
NCT02684123	Alopecia Areata	30 mg BID vs. placebo	Icahn School of Medicine at Mount Sinai	Phase II	Clinical efficacy and safety of 52 weeks therapy
NCT02307513	Behcet's Syndrome	30 mg BID vs. placebo	Celgene Corp.	Phase III	Clinical efficacy and safety of up to 64 weeks therapy
NCT02289417	Ulcerative Colitis	30 mg BID, 40 mg BID vs. placebo	Celgene Corp.	Phase II	Clinical efficacy and safety of 104 weeks therapy
NCT01583374	Ankylosing Spondylitis	20 mg BID, 30 mg BID vs. placebo	Celgene Corp.	Phase III	Clinical efficacy and safety of 24 weeks therapy
NCT01285310	Rheumatoid Arthritis	20 mg BID, 30 mg BID vs. placebo	Celgene Corp.	Phase II	Clinical efficacy and safety of up to 1.5 years therapy
NCT01074502	Acne	20 mg BID vs. placebo	Celgene Corp.	Phase II	Clinical efficacy and safety of 12 weeks therapy
NCT01041625	Lichen Planus	20 mg BID vs. placebo	Celgene Corp.	Phase II	Clinical efficacy and safety of 12 weeks therapy
NCT00931242	Atopic Dermatitis	20 mg BID vs. placebo	Celgene Corp.	Phase II	Clinical efficacy and safety of 12 weeks therapy
NCT00889421	Uveitis	30 mg BID vs. placebo	Celgene Corp.	Phase II	Clinical efficacy and safety of 6 months therapy
NCT00869089	Prurigo Nodularis	30 mg BID vs. placebo	Celgene Corp.	Phase II	Clinical efficacy and safety of 24 weeks therapy
NCT00814632	Vulvodynia	20 mg BID vs. placebo	Celgene Corp.	Phase II	Clinical efficacy of 12 weeks therapy
NCT00708916	Discoid Lupus Erythematosus	20 mg BID vs. placebo	Celgene Corp.	Phase II	Clinical efficacy of 16 weeks therapy

*Data derived from https://clinicaltrials.gov/*.

### Crisaborole approved for the treatment of AD

Atopic dermatitis is another common inflammatory skin disorder characterized by thickness, lichenification, excoriated, and fibrotic papules, which is usually associated with pruritus (Tollefson et al., 2014). The pathogenesis of AD is complicated and multifactorial, which may include environmental factors, skin barrier dysfunction, genetic predisposition, and immune dysfunction. The primary function of skin is to prevent water loss and to restrict entry of irritants, allergens, and other skin pathogens. Recently, skin barrier dysfunction has been widely established to be pivotal in the progression of AD (Tollefson et al., 2014). Pruritus is another troublesome symptom in AD patients and is responsible for causing further injury to the skin barrier. Increasing studies indicated that overexpression and overactivity of PDE4 led to the production of inflammatory cytokines and imbalance of T cell activity and polarization, which cause skin inflammation and disease exacerbation in AD patients.

Crisaborole (5-(4-Cyanophenoxy)-2,3-dihydro-1-hydroxy-2,1-benzoxaborole, AN-2728, Eucrisa, Figure 3C) was licensed for topical treatment of AD on December 14, 2016 (Kailas, 2017; Paton, 2017). Unlike topical corticosteroids and calcineurin inhibitors, crisaborole is a non-steroidal compound possessing phenoxy benzoxaborole skeleton with boron that inhibits PDE4 activity (IC_50_ = 490 nM) and shows excellent anti-inflammatory effects both *in vitro* and *in vivo* (Akama et al., 2009). Crisaborole could inhibit the release of inflammatory cytokines such as TNF-α, IL-1β, and IL-6 under the stimulation of PBMCs and macrophages derived from the human monocytic THP-1 cells (Freund et al., 2007). The animal toxicity studies were conducted following the systemic and topical administration of crisaborole to shrews, ferrets, rats, mice, and minipigs, which indicated that crisaborole had a wide safety margin and was available for further application in clinical trials (Cheape and Murrell, 2017). The 2-year animal carcinogenicity results showed that topical treatment with 2, 5, 7% crisaborle ointment in mice once daily was not tumorigenic without any change in the events of neoplastic microscopic lesions. Moreover, 300 mg/kg per day of crisaborole that was orally administrated to female rats could upregulate the incidence of benign granular cell populations in the distal reproductive tract, whereas it did not result in moribundity or death (Ciaravino et al., 2017).

Clinical trials of crisaborole dosing regiments demonstrated that treatment with 2% ointment twice daily was the greatest effective strategy in alleviating symptom severity when compared with the vehicle-controlled study (Stein Gold et al., 2015; Cheape and Murrell, 2017). Pruritus could verifiably be improved within a 1 week treatment of crisaborole (Draelos et al., 2016). Unlike systemic treatment, topical therapy of crisaborole failed to cause gastrointestinal adverse effects. Pharmacokinetics studies showed that topical application of crisaborole was rapidly absorbed and metabolized into two mainly established inactive metabolites (AN-7602 and AN-8323), which decreased the risk of unwanted systemic adverse effects (Zane et al., 2016a). In human healthy volunteers, there were no changes in vital signs and no crisaborole-related serious adverse events (Zane et al., 2016b). There were no reports about crisaborole-related serious treatment emergent adverse events (TEAEs), and in both the placebo and crisaborole groups, the rate of withdrawal from treatment because of TEAEs was 1.2% (Cheape and Murrell, 2017). A phase Ib, open-label, maximal-use study provided evidence that 2% crisaborle cream application was well-tolerated with lower systemic exposure in the first week in patients aged 2 years and older (Zane et al., 2016c). Following this, a phase IIa, bilateral, randomized, double-blind, 6-week study demonstrated that a total of 29 adverse events were observed in 11 of the 25 enrolled patients, and 90% of the patients were minor-to-mild and unrelated to therapy (Murrell et al., 2015). Moreover, two identically designed, vehicle-controlled, double-blind studies suggested that crisaborole-treated patients manifested great improvement in Investigator's Static Global Assessment (ISGA) score, and treatment-associated side effects were mild-to-moderate (Paller et al., 2016). Recently, the long-term safety observation from a 48-week, open-label, multicentric study in mild-to-moderate AD patients (*n* = 517) aged over 2 years showed that ≥1 TEAEs appeared in about 65% of the patients; however, the symptoms were mild or moderate and considered to be unrelated to treatment (93.1%) (Eichenfield et al., 2017). Although current cognition suggests that 2% crisaborole is a safe and efficacious drug for AD patients, long-term efficacy and whether it is safe for AD patients under the age of 2 years is unknown. Furthermore, there is not, yet, enough evidence to prove that it is safer or more effective than other existing topical treatment (Cheape and Murrell, 2017).

## PDE4 inhibitors under development for the treatment of inflammatory diseases

Over the last few years, PDE4 inhibitors have gratifyingly provided an alternative effective strategy for patients with inflammatory diseases. Though the adverse effects appear to be inevitable in clinical application, in addition to roflumilast, apremilast, and crisaborole, a series of novel PDE4 inhibitors have been designed to balance the therapeutic efficacy by minimizing the adverse effects. In this review, we presented the promising PDE4 inhibitors in practice for the treatment of inflammatory diseases, such as inflammatory, pulmonary, dermatological, and neurological diseases.

### PDE4 inhibitors developed for the treatment of inflammatory airway diseases

The pulmonary immune system is an obligatory coordinator that maintains the functions and integrity of the respiratory epithelium, which plays a vital role in defense against inhaled particles, virus, bacteria, and other foreign antigens (Gohy et al., 2016). However, pathological factors could break down its normal physiological functions and result in inflammation in the local pulmonary system or even systemic inflammatory responses (Parikh and Chakraborti, 2016). Phosphodiesterase-4 serves as an important modulator of airway inflammation; PDE4 inhibitors have been selected as an effective approach toward prevention of airway inflammation (Michalski et al., 2012). In addition to roflumilast, a series of PDE4 inhibitors, such as ronomilast, oglemilast, GSK256066, CHF6001, YM976, GS-5759, etc., have been in development (as shown in Table 2) (Beghè et al., 2013).

**Table 2:** Phosphodiesterase-4 (PDE4) inhibitors under development for the treatment of inflammatory diseases.

**Indication**	**Compound**	**Chemical structure**	**PDE4 inhibition (IC_50_)**	**Company**	**Clinical stage**	**NCT number**	**References**
Inflammatory Airway Diseases	CHF6001	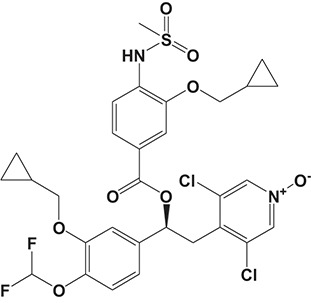	0.026 nM	Chiesi Farmaceutici	Phase II	NCT02986321	Moretto et al., 2015
	Ronomilast	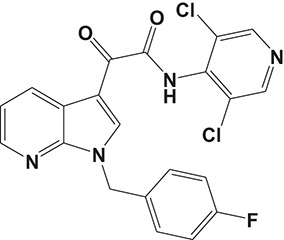	2 nM	Biotie	Phase I	NCT00977886	Parikh and Chakraborti, 2016
	Oglemilast	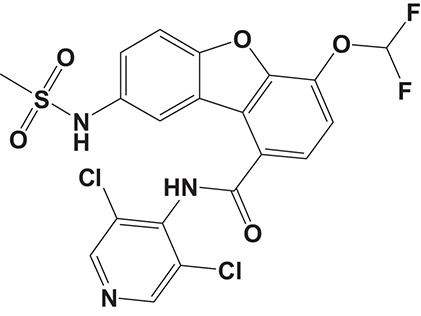	0.3 nM	Forest Laboratories	Phase II	NCT00322686	Tenor et al., 2011
	GSK256066	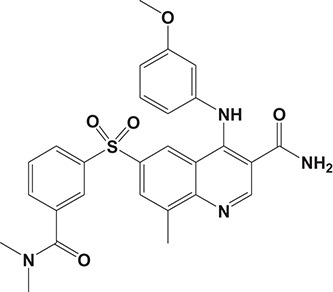	3.2 pM	GSK	Phase II	NCT00549679	Tralau-Stewart et al., 2011
	YM976	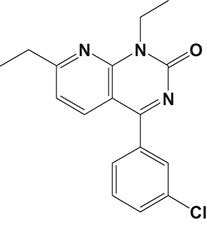	2.2 nM	Astellas	Phase I	—^a^	Aoki et al., 2000
	GS-5759	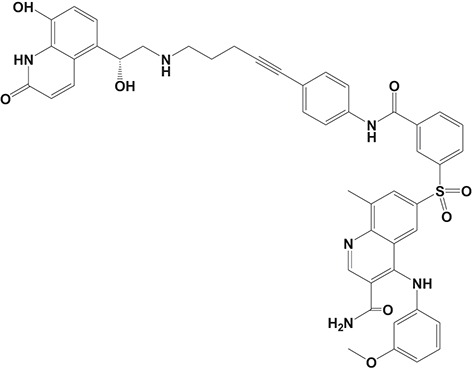	5 nM	Gilead	Preclinical	—	Tannheimer et al., 2014; Joshi et al., 2017
	GPD-1116	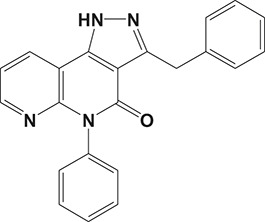	32 nM	ASKA Pharmaceutical	Phase II	—	Nose et al., 2016
	MEM1414	Undisclosed^b^	20 nM	Roche	Phase II	—	Leaker et al., 2014
	RPL554	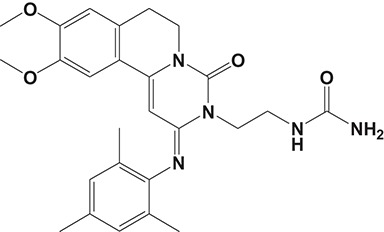	400 nM	Verona Pharma	Phase II	NCT03443414	Boswell-Smith et al., 2006
	ASP3258	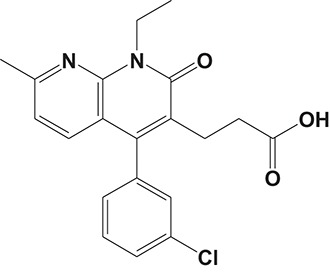	0.28 nM	Astellas	Preclinical	—	Kobayashi et al., 2011, 2012
Atopic Dermatitis	E6005	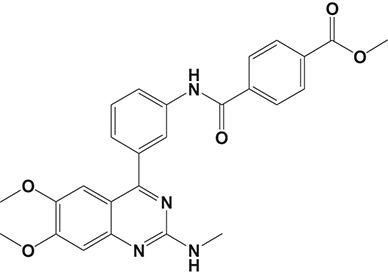	2.8 nM	Dermavant Sciences	Phase II	NCT01461941	Furue et al., 2017
	GW842470X	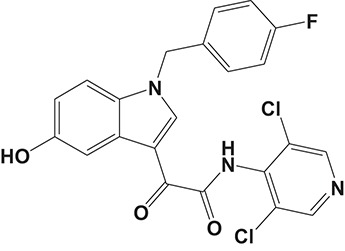	9.7 nM	GSK	Phase I	NCT00356642	Bäumer et al., 2007
	OPA-15406	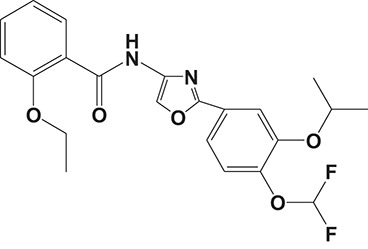	11.2 nM	Otsuka	Phase I	NCT03018691	Hanifin et al., 2016
	Leo-29102	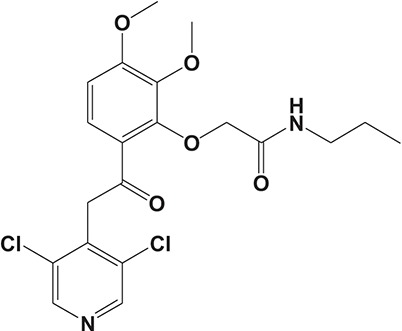	5 nM	LEO Pharma	Phase II	NCT01037881	Felding et al., 2014
	DRM02	Undisclosed	—	Dermira	Phase II	NCT01993433	Ahluwalia et al., 2017
Psoriasis	Pefcalcitol	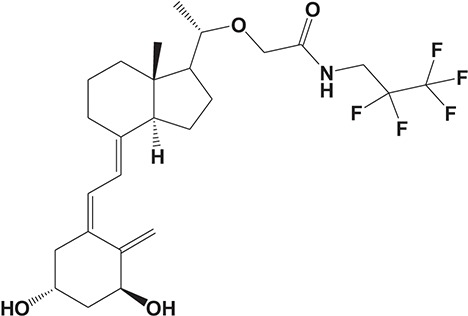	—	Maruho	Phase III	NCT01908595	Rizvi et al., 2015; Takeiri et al., 2017
	HFP034	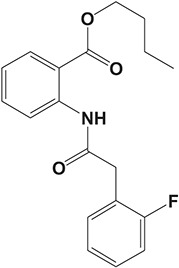	4.2 μM	Chang Gung University	Preclinical	—	Cheng et al., 2011; Lin et al., 2018
Rheumatoid Arthritis	CBS3595	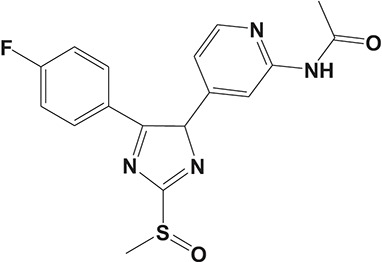	200 nM	c-a-i-r Biosciences GmbH	Phase I	—	Koch et al., 2014
	MK0873	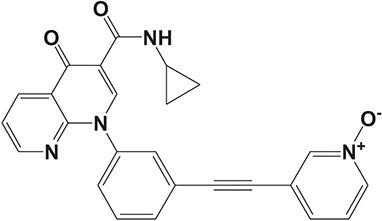	6.7 nM	Merck	Phase II	NCT00132769	Boot et al., 2008; Guay et al., 2008
	Revamilast	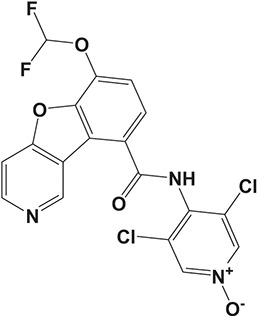	3 nM	Glenmark	Phase II	NCT01430507	Balasubramanian et al., 2016
Lupus	NCS 613	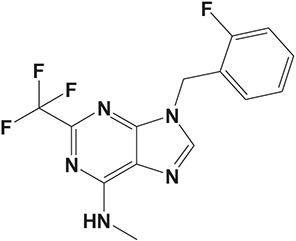	42 nM	GSK	Preclinical	—	Yougbare et al., 2011; Yougbaré et al., 2013
Neuroinflammation	FCPR03	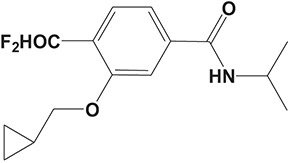	60 nM	Southern Medical University	Preclinical	—	Zou et al., 2017; Yu et al., 2018
	HT-0712	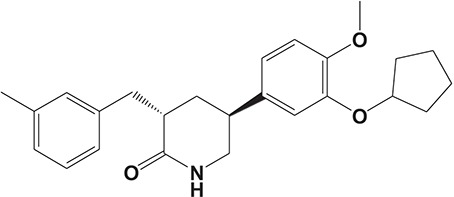	150 nM	Dart NeuroScience	Phase II	NCT02013310	Peters et al., 2014
	MK0952	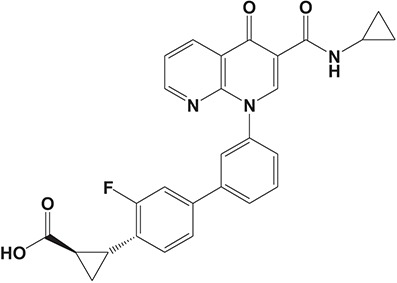	0.6 nM	Merck	Phase II	NCT00362024	Gallant et al., 2010
	ABI-4	Undisclosed	14 nM	Pfizer	Phase I	NCT02539550	Hedde et al., 2017
Liver Diseases	ASP9831	Undisclosed	—	Astellas	Phase II	NCT00668070	Ratziu et al., 2014

a *Unpublished or unreported*.

b *Chemical structure not open for public*.

#### CHF6001

The drug CHF6001, (S)-3,5-dichloro-4-(2-(3-(cyclopropylmethoxy)-4-(difluoromethoxy) phenyl)-2-(3-(cyclopropylmethoxy)-4-(methylsulfonamido) enzoyloxy) ethyl) pyridine 1-oxide, identified by Chiesi Farmaceutici S.p.A., was designed for the treatment of COPD and asthma via inhaled administration. The functional assays have demonstrated that CHF6001 was more potent than roflumilast and cilomilast in PDE4 inhibition and consequently inhibited the release of cytokines from human PBMCs, THP-1 cells, and murine RAW264.7 cells (Moretto et al., 2015). Moreover, CHF6001 dramatically suppressed the chemotaxis of eosinophils and the activation of oxidative burst in neutrophils as well as the production of cytokines from anti-CD3/CD28-induced CD4^+^ T cells, which revealed the therapeutic efficacy in pulmonary inflammation in ovalbumin-sensitized Brown Norway rats (Moretto et al., 2015; Villetti et al., 2015).

A double-blind, placebo-controlled, crossover study showed that compared with placebo, 400 or 1,200 μg of CHF6001 significantly attenuated the late asthmatic response (LAR) and resulted in prominent reduction in sputum eosinophil counts (Singh et al., 2016). When topically inhaled by ferrets and rats, no-observed-adverse-effect level dose was extremely lower than those observed for other PDE4 inhibitors (Villetti et al., 2015). Furthermore, the absorption, distribution, and elimination studies in rodents showed that MDR1, possibly BCRP, was vital for the disposition of CHF6001 and prevented the potential penetration in brain (Cenacchi et al., 2018). In summary, CHF6001 appeared to be devoid of typical emetic effects of PDE4 inhibitors in humans and showed an improved therapeutic index, which could account for limited systemic exposure and redundant brain penetration.

#### GSK256066

The drug GSK256066 is a slow but tight binding PDE4B inhibitor with dramatic activity (IC_50_ = 3.2 pM), which is more potent than any of the previously discovered compounds. *In vitro*, GSK256066 showed highly selective inhibition of PDE4 vs. PDE1, PDE2, PDE3, PDE5, PDE6, and PDE7 and inhibited inflammatory responses by LPS-stimulated human PBMCs and whole blood (Tralau-Stewart et al., 2011). Consistently, inhaled administration of GSK256066 to rats significantly suppressed pulmonary neutrophilia, indicating that it is an effective treatment for pulmonary inflammation. Preclinical investigations demonstrated that GSK256066 displayed therapeutic effects in acute pulmonary inflammation models induced by LPS and OVA, respectively (Nials et al., 2011; Tralau-Stewart et al., 2011).

In a randomized, double-blind, crossover study, GSK256066 displayed a considerable protective effect on the early asthmatic responses (EARs) and LARs of trachea inflammation. Inhalation of GSK256066 was proved to be well tolerated with limited systemic exposure, and the plasma levels of GSK256066 were unmeasurable after 4 h in majority of the individuals, which may obtain better efficacies by minimizing the potential for side effects (Singh et al., 2010). Additionally, a phase IIa, randomized, double-blind, multicenter, parallel-group, placebo-controlled, 4-week study with two dose interventions (25 and 87.5 mg) of GSK256066 indicated that the incidence rates of gastrointestinal side effects were neglectable in all therapy groups, and there were no vast changes in the inflammatory markers in the GSK256066 treatment group as well (Singh et al., 2010; Watz et al., 2013). However, further clinical studies are essential to confirm the favorable therapeutic index and safety profile of GSK256066.

### PDE4 inhibitors developed for the treatment of IBD

Patients suffering from Crohn's diseases (CD) and UC mostly manifest with severe diarrhea, fecal urgency, body weight loss, hematochezia, incontinence, and abnormal pain and even with fever caused by plethoric inflammation in the gut that lasts for weeks to months (Kumar et al., 2013). The inflammation in IBD patients is closely related to the increase in the production of pro-inflammatory cytokines. The activation of TNF-α and NF-κB are speculated to be linked to the injury of gut barrier integrity, contributing to breakdown of the extracellular matrix and multifocal ulcer formation. Given the suppression of TNF-α and NF-κB associated with PDE4 inhibition, certain PDE4 inhibitors, such as rolipram, mesopram, roflumilast, and apremilast, have been tested for the prevention of disease processes (Salari and Abdollahi, 2012; Kumar et al., 2013). Rolipram could ameliorate the clinical severity of acute inflammatory responses in 2,4,6-trinitrobenzenesulfonic acid (TNBS)-induced experimental colitis and also showed efficacy of both prevention and treatment of DSS-induced UC (Hartmann et al., 2000; Videla et al., 2006). Tetomilast, another PDE4 specific inhibitor, was shown to inhibit the release of inflammatory mediators from activated human leukocytes and to alleviate the fundamentally observed symptoms, production of inflammatory cytokines, and colon histological scores in the IL-10-deficient colitis mice (Ichikawa et al., 2008). However, owing to the serious adverse effects, further investigations of the effect of PDE4 inhibitors in IBD have been slower than those for inflammatory airway and skin diseases (Bickston et al., 2012; Spadaccini et al., 2017).

A previous study demonstrated that apremilast could reduce the production of TNF-α and matrix metalloproteinase-3 (MMP-3) in the gut lamina propria mononuclear cells (LPMCs) derived from the intestinal mucosa, which might lead to the promotion of mucosal healing and represent an oral agent in patients with IBD (Gordon et al., 2009). In addition to psoriasis and PsA, apremilast was intended to be an attractive candidate for patients with IBD. A phase II, randomized, placebo-controlled study (NCT02289417) was designed to investigate the efficacy and safety of apremilast (30 and 40 mg BID) for the treatment of patients with active UC. There were no clinical data posted about this study.

### PDE4 inhibitors developed for the treatment of AD

Historically, therapeutic options for AD patients have been subjected to topical usage of corticosteroids and calcineurin inhibitors or have been referred to the systemic immunosuppressive agents and phototherapy. In clinical trials, unexpected adverse events exist following these therapeutics; therefore, more therapeutic strategies with limited cutaneous and systemic adverse effects are desired for AD patients (Zebda and Paller, 2018). Targeting with PDE4 inhibitors contributes to the reduction of inflammatory cytokines and chemokines in psoriatic skin. The 2% crisaborole ointment is the only PDE4 inhibitor that has been approved for adults and children older than 2 years suffering from AD. It is worth mentioning that many other PDE4 inhibitors have also emerged in clinical trials with promising therapeutic efficacy, including E6005/RVT-501, OPA-15406, Leo-29102, GW842470X, etc.

#### E6005/RVT-501

The drug E6005, also named as RVT-501, could specifically inhibit human PDE4 activity (IC_50_ = 2.8 nM) and modulate varieties of cytokine production from lymphocytes and monocytes. In the murine AD models, topical application of E6005 ointment ameliorated the clinical manifestations of allergic dermatitis-like skin injury via elevation of cAMP and nerve growth factor (NGF) in the skin (Ishii et al., 2013, 2014). In NC/Nga mice, topical application of E6005 could apparently suppress the behavior of spontaneous hind-paw scratching, the spontaneous activity of cutaneous nerve, and an itch-associated response (Andoh and Kuraishi, 2014; Andoh et al., 2014). Unlike treatment with steroids, topical application of E6005 improves skin inflammation and pruritus of pediatric and adult AD patients without any serious side effects (Furue et al., 2014, 2017). In a randomized, double-blinded, vehicle-controlled, and multiple ascending dose study, the skin lesion severity scores were significantly reduced in a dose-dependent manner (Ohba et al., 2016a). Moreover, topical treatment with E6005 led to rapid clearance from the peripheral blood system and limited penetration to brain, which suggested that E6005 treatment might be a promising and safe therapeutic strategy for AD patients (Ohba et al., 2016b).

#### OPA-15406

The drug OPA-15406 acts as a PDE4 inhibitor with high selectivity for PDE4B, reducing severe inflammation in the local skin lesions by suppressing the expression of chemical mediators that are believed to exacerbate the clinical signs of allergic dermatitis. Levels of OPA-15406 in the blood were negligible following topical treatment, contributing to mild systemic adverse events (Hanifin et al., 2016; Zebda and Paller, 2018). A phase I trial in healthy adult donors indicated that there are no adverse events in the 2-week treatment by applying 0.3, 1, or 3% formulation of 5 g OPA-15406 ointment to a 1,000 cm^2^ skin area. A phase II, double-blind, randomized, vehicle-controlled study was designed to assess the efficacy and tolerability of topical treatment with OPA-15406 in AD patients. The 1% OPA-15406 ointment significantly improved the pruritus score and the severity index score with low incidence of adverse events (Hanifin et al., 2016; Eichenfield and Stein Gold, 2017).

#### LEO-29102

Leo-29102 was developed by Leo Pharma in 2014, and it acts as a potent and selective PDE4 inhibitor with excellent anti-inflammatory properties (Felding et al., 2014; Bonnel et al., 2018). By this point, a phase II study with Leo-29102 had been launched, and the study intended to observe the clinically relevant efficacy of the drug in treating AD. There is a phase II dose finding trial that compares five dose strengths in mild-to-moderate AD patients with vehicle in a 4-week, twice daily treatment manner. Briefly, the group treated with all five doses showed a numerically better effect than the vehicle on the eczema area and severity index (EASI) score from baseline to end of therapy. Moreover, analysis showed dose-dependent statistically significant effects of Leo-29102 on the patient's assessment of pruritus and overall assessment of disease severity (Zebda and Paller, 2018).

### PDE4 inhibitors developed for the treatment of psoriasis

Polytype psoriasis affects a reasonably small population worldwide. Plaque psoriasis, further referred to as psoriasis vulgaris with erythematous and indurated plaques, is the most common manifestation in psoriasis patients (Greb et al., 2016). A range of therapeutic strategies are available for the treatment of patients with psoriasis, including topical immunosuppressive agents, photo-based therapies, and emerging biologic agents. More recently, numerous biologic agents that target pro-inflammatory cytokines have been well-established and novel therapies, such as PDE4 inhibitors, have been also developed (Lønnberg et al., 2014). Following in the footsteps of the treatment with apremilast, pefcalcitol is currently in phase III clinical trials for plaque psoriasis, and HFP034 has been developed under preclinical stage for psoriasis treatment.

#### Pefcalcitol

Pefcalcitol (also known as M5181), an analog of vitamin D_3_ developed by Maruho Pharmaceutical, is an anti-psoriatic drug candidate with promising PDE4 inhibitory activity (Takeiri et al., 2017). Preclinical studies suggested that topical application of pefcalcitol was an effective treatment for plaque psoriasis with fewer side effects than vitamin D_3_. Recently, the phase III clinical 8-week trial has been designed to estimate the efficacy and safety profile of treatment with pefcalcitol ointment in a larger population of patients with stable plaque psoriasis.

#### HFP034

The drug HFP034, butyl 2-[2-(2-fluorophenyl) acetamido] benzoate, was derived from anthranilic acid derivatives and exhibited potent inhibitory activity on PDE4 enzyme (IC_50_ = 4.2 μM) and fMLP-induced generation of ${\text{O}}_{2}^{\cdot -}$ in human neutrophils *in vitro* (Cheng et al., 2011). The drug HFP034 was found to be well-absorbed into the skin and, meanwhile, causes negligible irritation on healthy skin. In the imiquimod-induced mouse psoriasis model, topical application of HFP034 could ameliorate the skin lesions and epidermal thickness via inhibition of inflammatory cytokines and chemokines and infiltration of neutrophils. Further investigations suggested that HFP034 increased the skin concentration of cAMP and inhibited the NF-κB activity (Lin et al., 2018). These results indicate that topical application of HFP034 tends to be a potential therapeutic option for psoriasis patients.

### PDE4 inhibitors developed for the treatment of RA

Rheumatoic arthritis is a chronic, relapsed inflammatory autoimmune disease and is primarily related to abnormal articular inflammation, synovial joint injury, autoantibodies production, and increased disability incidence. Following the erosion of cartilage and bone, local upregulation of inflammatory cytokines and chemokines results in vast infiltration of immune cells (McInnes et al., 2016). In the past two decades, monoclonal neutralizing-antibodies that target TNF-α, IL-1, IL-6, and IL-17 have widely been used as biological therapy. The first and most frequently used monoclonal antibody is TNF-α inhibitor, infliximab (Koenders and van den Berg, 2015). As an alternative therapy in RA patients, PDE4 inhibitors, such as apremilast and revamilast, showed dramatic effects on reducing the secretion of TNF-α. To some extent, given the price and adverse events, PDE4 inhibitors are much more available than monoclonal antibodies for RA.

#### Revamilast

Revamilast (also known as GRC 4039) was developed as an orally active PDE4 inhibitor by Glenmark, which was under development for the treatment of RA, plaque psoriasis, asthma, and other inflammatory disorders. Revamilast has successfully finished preclinical and phase I trials for RA treatment. The pharmacodynamic evaluation study showed a significant inhibition of the inflammatory marker TNF-α in healthy human volunteers treated with revamilast. A phase IIb, randomized, double-blind, parallel group, placebo-controlled, 12-week study was conducted to evaluate the efficacy, safety, and tolerability of RA patients. The primary objective was to record the population of patients that achieve ACR20 responses at 12 weeks, and the secondary objective consisted of determining the percentage of patients with ACR50 and ACR70 responses, changing the DAS-28 score and serum CRP and ESR levels as well as the frequency and use of rescue medication. However, no results were reported about the clinical trial.

### PDE4 inhibitors developed for the treatment of lupus

Lupus is an autoimmune disease with painful and swollen joints, swollen lymph nodes, and a red rash symptom. The etiology of lupus is multifactorial and includes contributions from the environment, stochastic factors, and genetic susceptibility (Kuhn et al., 2016; Tsokos et al., 2016). Keravis et al. found that PDE4 was identified as the main intracellular enzyme for cAMP hydrolysis in the kidney of CBA/J mice, whereas PDE2 and PDE3 showed a lower extent of cAMP and cGMP hydrolysis property. Correspondingly, there is a significant increase in the cAMP-specific PDE activity in the kidney of 18-week-old MRL/*lpr* mice (Keravis et al., 2012). To date, selective PDE4 inhibitors have been tested in lupus treatment, such as apremilast and NCS613.

#### NCS613

The drug NCS613 displayed anti-inflammatory properties, providing an alternative or complementary option for the management of systemic lupus erythematosus (SLE) (Yougbare et al., 2011; Kuhn et al., 2016). A recent study in MRL/*lpr* lupus-prone mice revealed that NCS613 effectively decreased proteinuria and increased the survival rate of MRL/*lpr* mice. The drug NCS613 also inhibited basal and LPS-induced TNF-α secretion from PBMCs of lupus patients, which was observed in MRL/*lpr* mice as well (Keravis et al., 2012). In another study, NCS613 downregulated PDE4B and, meanwhile, upregulated PDE4C expression was observed in PBMCs from human healthy individuals and SLE patients. Moreover, following LPS induction, NCS613 reduced the secretion of TNF-α, IL-6, and IL-8 owing mainly to the abolishment of phosphorylation levels of p38 MAPK and the NF-κB translocation (Yougbaré et al., 2013).

### PDE4 inhibitors developed for the treatment of BS

Behcet's syndrome was first identified in 1937 by Behcet with its great prevalence in the Middle East, and further studies showed that BS was characterized by multiple mucocutaneous manifestations, including aphthae, erythema nodosum, genital ulcers, skin lesions, uveitis, and thrombophlebitis (Leccese et al., 2018). Although the pathogenesis of BS is currently unclear, intractable inflammation has been considered as the main concept in the progression of BS. Several immunomodulatory and immunosuppressive agents showed some beneficial effects in approving the mucocutaneous lesion and other symptoms. In addition, IL-1 inhibitors, TNF-α inhibitors, and PDE4 inhibitors have also proven to be effective therapeutic strategies in the treatment of BS (Vitale et al., 2016).

Apremilast has been subjected to development for BS patients. In the phase 2 randomized, double-blind placebo controlled, parallel group study (NCT00866359), apremilast met the primary and secondary end points at the end of 12 weeks. The results demonstrated that patients who received apremilast showed a rapid onset of efficacy within 2 weeks and a striking maintenance along with drug treatment. Apremilast significantly approved the disease activity and quality of life measures with lower oral ulcers, greater baseline of oral ulcer pain, and fewer genital ulcers than those observed in the placebo group (Hatemi and Yazici, 2017). Moreover, the adverse effects observed in the apremilast group were similar to those in the previous clinical studies (Hatemi et al., 2015). Recently, Celgene Corporation has presented the data of phase 3 randomized study, followed by an active-treatment phase in the subjects with active BS at the 2018 American Academy of Dermatology (AAD) Annual Meeting. The observations suggested that 30 mg of apremilast twice daily statistically significantly reduced the number of oral ulcers, oral ulcer pain, and improved overall disease activity. Altogether, apremilast appears to be a better management for such patients and further application in other manifestations of BS is required to estimate the efficacy and safety of apremilast (Hatemi and Yazici, 2017).

### PDE4 inhibitors developed for the treatment of neuroinflammation

Accumulating evidence indicated that neuroinflammation largely initiates and exacerbates the process of nervous system diseases, including depression, Alzheimer's disease, multiple sclerosis, etc. (Heneka et al., 2015). Functioning as an important regulator of cAMP, PDE4, is highly expressed in the brain (Maurice et al., 2014). Advancements in PDE4-targeted therapy have shown promise in neuroinflammation-related diseases. Rolipram is the first blood-brain-barrier (BBB) permeable PDE4 inhibitor, developed for ameliorating neuroinflammation. Rolipram has been proven to be effective in various animal models, including depression, neuropathic pain, Alzheimer's disease, Parkinson's disease, and multiple sclerosis (Rose et al., 2005; García-Osta et al., 2012; Pearse and Hughes, 2016). However, the clinical application of rolipram is limited because of its behavioral and other severe side effects (Zhu et al., 2001). Following the discovery of rolipram, more selective inhibitors have been developed in practice, such as FCPR03, MK0952, HT-0712, ABI-4.

#### FCPR03

The drug FCPR03, a novel selective PDE4 inhibitor, was designed for depression and neurodegenerative diseases. *In vitro*, FCPR03 inhibited the immune responses in LPS-stimulated BV-2 cells, a microglial cell line, and its anti-inflammatory effects could be blocked by a PKA inhibitor, H89. *In vivo*, FCPR03 suppressed pro-inflammatory mediators by increasing the level of cAMP, promoting CREB phosphorylation, and inhibiting NF-κB activation in the cortex and hippocampus of LPS-immunized mice (Zou et al., 2017). Furthermore, a study in mice challenged by LPS found that 1 mg/kg/day FCPR03 showed anti-depressant effects as confirmed by the decrease in depressant-like behaviors with the duration of immobility in the forced swim and tail suspension tests (Yu et al., 2018). In summary, FCPR03 is a potential drug candidate for the treatment of depression, and further clinical trials should be performed to confirm its effects in patients.

### PDE4 inhibitors developed for the treatment of liver diseases

Hepatic steatosis unassociated with alcohol affects up to 25% of the world's population. Nonalcoholic steatohepatitis (NASH) develops in patients who are not alcoholic and leads to liver damage that is histologically indistinguishable from alcoholic hepatitis. Nonalcoholic steatohepatitis is characterized by hepatocellular injury, innate immune cell-mediated inflammation, and progressive liver fibrosis (Ibrahim et al., 2018).

#### ASP9831

The drug ASP9831, developed by Astellas Pharma Inc. for the treatment of NASH, exhibited potent anti-inflammatory and antifibrotic effects in preclinical studies. A phase II, randomized, double-blind, placebo-controlled, 12-week treatment study aimed to determine the effects in NASH patients by assessing clinical signs, laboratory data, and biomarkers. However, oral administration of ASP9831 failed to significantly alter the biochemical markers, ALT and AST, in clinical trials. The gastrointestinal disorders occurred more frequently, and the most adverse events in the ASP9831 groups were mild (Ratziu et al., 2014). There exist the difficulties of developing PDE4 inhibitor for NASH, and more extensive efforts are required to find potential drug candidates.

## Perspectives

Enhancing intracellular cAMP levels by inhibiting PDE4 has been proven to be an available therapeutic strategy to alleviate several diseases in which inflammation plays a critical role, such as pulmonary, dermatological, and severe neurological diseases (Hernández-Flórez and Valor, 2016). Nonselective PDE inhibitors, including theophylline, ibudilast, and doxofylline, were discovered during early development. Later, beginning with the first selective PDE4 inhibitor, rolipram, several other active molecules emerged on the road to the discovery of novel PDE4 inhibitors (Martinez and Gil, 2014). Fortunately, roflumilast is the first approved drug of this class for the treatment to reduce the risk of COPD exacerbations in patients with severe COPD associated with chronic bronchitis and a history of exacerbations (Kawamatawong, 2017). Apremilast, the second PDE4 inhibitor on the marker, was licensed in 2014 for adult patients with active PsA and patients with moderate-to-severe plaque psoriasis who were candidates for phototherapy or systemic therapy (Chiricozzi et al., 2016). Apremilast was launched at a 30% discount to the average price of biologic drugs. In the United States, apremilast has managed to be a leader in the new-to-brand segment, and it accounts for a 42% share in the psoriasis segment. Another PDE4 inhibitor, 2% crisaborole ointment, was approved in 2016 for topical treatment of mild-to-moderate atopic dermatitis in patients aged 2 years and older (Paton, 2017). Nevertheless, various adverse effects related to the application of PDE4 inhibitors are the primary bottleneck in new drug development (Matera et al., 2014).

To minimize the adverse effects of PDE4 inhibitors, there may be three improvement strategies:

1. Design potent isoform-specific inhibitors or allosteric modulators. Numerous PDE4 inhibitors have been impeded in clinical development by the side effects of gastrointestinal effects or neurological disorders, observed both in humans and various animal species. Phospodiesterase-4 is presented by four subtypes, PDE4A, 4B, 4C, and 4D and each contains upstream conserved region 1 (UCR1) and UCR2. In the brain, PDE4A, PDE4B, and PDE4D, excluding PDE4C, are extensively expressed, which is speculated to be associated with reward and affect. Currently, most PDE4 inhibitors are available for the inhibition all four subtypes and for the alteration of the cAMP concentrations beyond normal physiological levels (Burgin et al., 2010). In addition, PDE4 plays a critical role in anesthesia induced by α_2_-adrenoceptor activation. Robichaud et al. reported that shorter sleeping time was observed in PDE4D-deficient mice, but not in PDE4B-deficient mice, than wide-type mice under xylazine/ketamine-induced anesthesia, a behavioral observation model of emesis in non-vomiting species. The findings indicated PDE4D, but not PDE4B, could modulate the activity of α_2_-adrenoceptor, which suggests PDE4D inhibition is most likely responsible for emesis and other side effects (Robichaud et al., 2002). Thus, isoform-specific PDE4 inhibitors might be more effective therapeutics (Pagès et al., 2009; Page, 2014). Recently, X-ray crystallography has identified the binding modes for several classes of PDE4 inhibitors, which allows the design of more potent compounds than before (Xu et al., 2000). Jansen et al. presented a systemic structure chemogenomics analysis in 2016 about a total of 220 PDE catalytic domain crystal structures in the protein data bank (PDB) focusing on PDE-ligand interaction, which provide a compressive map to guide the discovery of PDE4 inhibitors (Jansen et al., 2016). Alternatively, PDE4D has a phenylalanine (Phe) at the 196th position within UCR2, whereas a tyrosine at the 274th position in PDE4A, 4B, and 4C. Unlike the traditional competitive PDE4 inhibitors that completely inhibit enzyme activity, the UCR2-directed allosteric modulators function by weakening the interaction with the active site and, thus, only partially inhibit cAMP hydrolysis with the maximum inhibition over 50%, which are more likely to reduce the potential to cause emesis while maintaining the biological activity both *in vitro* and *in vivo* (Burgin et al., 2010; Gurney et al., 2011). The drug D159687, a negative allosteric modulator, bridges the Phe196 of PDE4D and does not cause emesis and nausea at 30 mg/kg (Titus et al., 2018). On the other hand, the emesis is caused in part by a noradrenergic pathway of area postrema and nucleus of the solitary tract and can be improved by limiting penetration to the brain. Although there remains a formidable challenge taking the evident similarities of the active sites of the various PDE4 subtypes into consideration, with the structural information available, patent isoform-specific inhibitors may be discovered in the future (Gavaldà and Roberts, 2013).
2. Change the routine of administration. Roflumilast was originally designed for oral administration, which inevitably had severe gastrointestinal adverse reactions and weight loss. In contrast to roflumilast, GSK256066, a highly specific inhibitor of PDE4B, was established for inhaled administration for airway inflammation with limited systemic exposure, which accounts for the fact that the gastrointestinal adverse reactions were mild in the treatment group (Tralau-Stewart et al., 2011). Additionally, crisaborole was topically applied for the treatment of skin inflammation, and clinical trials suggested that unlike systemic treatment of apremilast, topical application of 2% crisaborole ointment revealed great safety profile and did not cause significant gastrointestinal adverse effects, whereas oral administration of apremilast caused mild-to-moderate adverse effects such as nausea, diarrhea, and significant weight loss.
3. Combination therapy. The first-line bronchodilators that appear in the treatment of COPD consist of long-acting β2-adrenergic receptor agonists (LABA) and long-acting muscarinic agents (LAMA). Combination therapy has been widely used for its benefits over the monotherapies in the pulmonary function and other clinical outcomes such as dyspnea (Parikh and Chakraborti, 2016). The first LABA-LAMA combination QVA149 (indacaterol-glycopyrronium) is relatively safe with minimal or moderate adverse effects and has been approved for COPD treatment since 2013 (Thompson, 2013; Ficker et al., 2017). Accordingly, combining PDE4 inhibitors with various agonist/antagonists targeting adenylyl cyclase (AC), β-adrenoceptor, glucocorticoids, Ca^2+^ channel blocker, oligonucleotides, cytokines inhibitors, NO synthase inhibitors, and COX-2 inhibitors is being postulated as effective for the treatment of inflammatory airway diseases manifesting as LABA-LAMA effects (Parikh and Chakraborti, 2016). A previous investigation found that coadministrated roflumilast with some other medications, such as bronchodilators, could dramatically reduce the symptoms and disease exacerbations along with lower side effects than roflumilast treatment only (Calverley et al., 2009). The emetic responses could be overcome by the coadministration of the Ca^2+^ channel antagonist (CCA), which depolarize the L-type Ca^2+^ currents in the locus coeruleus neurons and also relax the lung smooth muscles (Wang and Wang, 2005; Wang and Cui, 2006).

## Author contributions

HL and WT wrote this manuscript. WT and JZ revised it critically for important intellectual contents.

### Conflict of interest statement

The authors declare that the research was conducted in the absence of any commercial or financial relationships that could be construed as a potential conflict of interest.
